# Is serum bilirubin level a predictor factor in parotid gland carcinoma?^☆^

**DOI:** 10.1016/j.bjorl.2019.01.013

**Published:** 2019-03-08

**Authors:** Suphi Bulğurcu, Mehmet İdil, İbrahim Çukurova

**Affiliations:** a Sultan Abdülhamid Han Training and Research Hospital, Department of Otorhinolaryngology, İstanbul, Turkey; b Tepecik Training and Research Hospital, Department of Otorhinolaryngology, Izmir, Turkey

**Keywords:** Bilirubin, Parotid gland, Survival, Bilirrubina, Glândula parótida, Sobrevida

## Abstract

**Introduction:**

Bilirubin levels have been associated with risk of several malignancies. The association between pretreatment serum bilirubin levels and overall survival of patients with parotid gland carcinoma is unclear.

**Objectives:**

In this study, we assessed the effect of serum bilirubin levels to overall survival in malignant parotid tumors.

**Methods:**

This study included a total of 35 patients, 15 female and 20 male. The mean age of these patients was 60.7 ± 14.5 years. All patients who were diagnosed with parotid gland carcinoma and underwent total parotidectomy between 2008 and 2018, were retrospectively assessed. The relationship between the overall survival of patients and total bilirubin, direct bilirubin, and indirect bilirubin levels was estimated. The receiver operating characteristic (ROC) curve analysis was performed to determine the optimal cut-off points.

**Results:**

Patients with low direct bilirubin, total bilirubin and indirect bilirubin had significantly longer overall survival than those with high levels. Cut-off values for total bilirubin, direct bilirubin and indirect bilirubin were detected as 0.545 mg/dL, 0.175 mg/dL and 0.435 mg/dL, respectively.

**Conclusion:**

In our study, we observed that increased preoperative bilirubin levels are associated with reduced survival time in the postoperative period of patients with parotid gland carcinoma.

## Resumo

**Introdução:**

Os níveis de bilirrubina têm sido associados ao risco de várias lesões malignas. A associação entre os níveis séricos de bilirrubina pré-tratamento e a sobrevida global dos pacientes com carcinoma da glândula parótida ainda não é clara.

**Objetivos:**

Neste estudo, avaliamos o efeito dos níveis séricos de bilirrubina na sobrevida global em tumores malignos de parótida.

**Método:**

Este estudo avaliou 35 pacientes, 15 do sexo feminino e 20 do masculino. A média de idade foi de 60,7 ± 14,5 anos. Pacientes que foram diagnosticados com carcinoma da glândula parótida e submetidos a parotidectomia total entre 2008 e 2018 foram avaliados retrospectivamente. A relação entre a sobrevida global dos pacientes e os níveis de bilirrubina total, bilirrubina direta e bilirrubina indireta foi estimada. A análise da curva *Receiver Operating Characteristic* foi realizada para determinar os pontos de corte ideais.

**Resultados:**

Pacientes com níveis mais baixos de bilirrubina direta, bilirrubina indireta e bilirrubina total tiveram sobrevida global significantemente maior do que aqueles com valores mais altos. Valores de corte para bilirrubina total, bilirrubina direta e bilirrubina indireta foram estabelecidos como 0,545 mg/dL, 0,175 mg/dL e 0,435 mg/dL, respectivamente.

**Conclusão:**

Em nosso estudo, verificamos que níveis de bilirrubina aumentados no pré-operatório estão associados à redução do tempo de sobrevida no período pós-operatório em pacientes com carcinoma da glândula parótida.

## Introduction

Parotid gland carcinoma is a rare tumor that accounts for approximately 0.5% of all carcinomas and represents less than 5% of all head and neck cancers.^1^ Due to several factors, it is not easy to assess the prognostic factors for parotid carcinoma or the survival rate; and these factors include low incidence of the disease, and the existence of various histopathological types and pathological grades.^2^

Bilirubin is metabolized from the liver through a normal catabolic pathway of vertebrates, in which it breaks down. In the liver, glucuronyltransferase enzyme conjugates bilirubin with glucuronic acid, and this reaction gives water solubility features to the bilirubin. The conjugated bilirubin is named direct bilirubin (DBIL). Similarly, unconjugated bilirubin, namely indirect bilirubin (IBIL), accounts for more than 80% of the total bilirubin. In the diagnosis of hepatobiliary and hematological diseases; bilirubin is accepted as an important marker. In addition to that, some studies have showed that bilirubin has an antioxidant characteristic and plays an important role in inflammation and cancer.^3^, ^4^ Furthermore, in vitro studies showed that bilirubin can induce apoptosis and suppress the proliferation of cells in many tumor types.^5^ In the light of these studies, serum bilirubin might have a protective or antitumor effect in human malignancies.^6^

The relationship between bilirubin levels and parotid gland carcinoma has not been studied previously. In this study, we assessed the effect of serum bilirubin levels to overall survival in malignant parotid tumors.

## Methods

In this study, a total of 35 patients, 15 female and 20 male were included. The mean age of these patients was 60.7 ± 14.5 years. All participants were diagnosed with parotid gland carcinoma and underwent total parotidectomy between November 2008 and February 2018. After the approval of the local ethics committee (2018/10-13), an informed consent of all patients was taken before the surgery, and their records were retrospectively investigated. Fine needle aspiration biopsy was performed preoperatively, and in all patients the masses were diagnosed as malignant. Preoperative computed tomography (Somatom Plus Volume; Siemens, Erlangen, Germany) was used to evaluate the size and possible metastasis foci. Bilirubin values were measured in the blood samples preoperatively from patients using an automated blood cell counter (Beckman Coulter analyzer, CA, United States) in the biochemical laboratory.

The relationship between the survival time after total parotidectomy and the preoperative serum levels of total bilirubin (TBIL), DBIL, IBIL was investigated and in addition to that, the effect of age, gender, tumor stage, lymph node involvement, histological differentiation on the survival rate was also investigated.

Patients with hepatobiliary and hematologic diseases, insufficient survival data or pre-treatment hematology test data, patients with distant metastasis and patients with surgical margin positivity were excluded from the study.

The lesions in parotid gland were evaluated according to the 7th edition of the American Joint Committee (AJCC) cancer staging manual.^7^ Serum levels of DBIL and TBIL were measured using the bilirubin kit (BioSino Bio-Technology & Science Inc., Beijing, China) and were analyzed by using the Automatic Biochemistry Analyzer (AU680, Beckman Coulter, Inc.).

In the analysis of the data, SPSS and Microsoft excel computer programs were used. For statistical analysis; descriptive analyzes (frequency distributions, mean, standard deviation) and Kolmogorov–Smirnov normality distribution were used. The *t*-test, Chi-square and Fisher's Exact test were used because the data were fit to normal distribution. Survival averages were assessed by Kaplan–Meier method and the effect of selected variables on survival were investigated with Cox regression analysis. The results were evaluated at 95% confidence interval with a significance level of *p* < 0.05.

## Results

**Figure 1:**
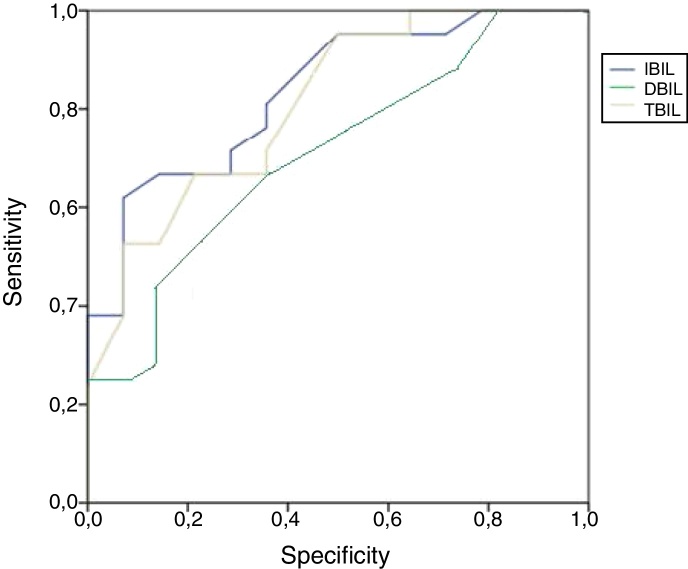
Area under the receiver operating characteristics curve for TBIL, DBIL and IBIL (0.835, 0.804, 0.862, respectively) (95% confidence interval).

According to the data, 21 patients were found to be alive and 14 patients were dead. The mean survival age of the patients was 70.49 ± 8.85 years. There was no statistically significant difference for overall survival period between the groups in terms of age and gender (*p* = 0.17 and *p* = 0.08, respectively). The baseline demographic features of patients with parotid gland carcinoma are displayed in Table 1. Patients with low TBIL, DBIL and IBIL had significantly longer overall survival than those with high TBIL, DBIL and IBIL (*p* = 0.008, *p* = 0.028 and *p* = 0.006, respectively). Cut-off values for TBIL, DBIL and IBIL were detected as 0.545 mg/dL, 0.175 mg/dL and 0.435 mg/dL, respectively (Fig. 1).

**Table 1:** Patients’ characteristics.

Variables	Number of patients	%
*Age*	Mean: 60.7/range: 35–88	
*Gender*		
Female	15	42.8
Male	20	57.2
*Histopathologic diagnosis*		
Mucoepidermoid	16	45.7
Epithelial–myoepithelial	6	17.1
Ex pleomorphic adenoma	5	14.2
Acinic cell	3	8.5
Ductal	3	8.5
Adenoid cystic	2	5.7
*Stage*		
I	5	14.2
II	7	20
III	8	22.8
IV	15	42.8
*N classification*		
*N* (+)	20	57.1
*N* (−)	15	42.8
*Histological differentiation*		
Well	15	42.8
Moderate	4	11.4
Poor	16	45.7
*Adjuvant therapy (KT* *±* *RT)*		
+	22	62.8
−	13	37.1

## Discussion

Assessing the prognostic factors for parotid carcinoma or the survival rate of patients is difficult due to several reasons. First of all, the disease has low incidence and present various histopathological sub-types and pathological grades. Secondly, the prevalence of low-grade malignancy carcinoma is high, requiring a long-term follow-up. Finally, in the course of this disease, maintaining a specific treatment plan for a particular diagnosis is also difficult.^2^, ^8^

There is a reduction in the level of bilirubin in patients with parotid gland carcinoma and this reduction might show the antioxidant, anti-inflammatory and anticancer effects of bilirubin. Inflammation can promote the progression of cancer. Bilirubin is able to inhibit this inflammatory process by preventing the migration of leukocytes into target tissues through the interruption of vascular cell adhesion molecule-1 (VCAM-1)-dependent cell signaling.^9^, ^10^, ^11^ The normal value of bilirubin is approximately stable and does not change with age. Additionally, diet and exercise merely affect the blood level of bilirubin. But corrupted liver functions can cause elevated bilirubin levels.^12^ Therefore, patients with hepatobiliary disease were excluded from the study.

In a study of Gao et al., with 469 patients; elevated serum direct bilirubin concentration was related with poor prognosis in rectal cancers, they determined the cut-off value of DBIL as 0.26 mg/dL.^13^ In a study of Zhang et al., with 986 patients; it was found that elevated DBIL level was associated with poor postoperative outcomes in colorectal cancer patients with staged 2 and 3 diseases, and they determined the cut-off value of DBIL as 0.36 mg/dL.^6^ In a study of Zhang et al., conducted with 173 patients, high TBIL, DBIL and serum liver enzymes were found to be independent prognostic factors in patients with intrahepatic cholangiocarcinoma.^14^ Li et al., carried out a study with 1617 patients and reported that moderately elevated bilirubin levels before treatment were associated with prolonged overall survival in patients with resected non-small-cell lung cancer, and cut-off DBIL value was detected as 0.345 mg/dL.^15^

Thus, it is obvious that various markers can be found for predicting the survival rate of different cancer types, but contrary statistical analyses may also occur for these different results. In our study, we found that patients with lower DBIL, TBIL and IBIL had significantly longer overall survival than those with higher DBIL, TBIL and IBIL. Cut-off values for TBIL, DBIL and IBIL were detected as 0.545 mg/dL, 0.175 mg/dL and 0.435 mg/dL, respectively.

Surely, this retrospective study has some limitations. First of all, since parotid gland carcinomas are rare, studies with larger patient populations will yield more reliable results. In addition, our study has histopathological variability and more specific bilirubin values can be obtained with a study which is conducted with a patient population who has same histopathological diagnosis. Preoperative hepatobiliary disease was not observed in our studied patients. However, there were patients with history of alcohol ingestion in their past. The alcohol is partially responsible for liver function damage and this damage can also affect the serum levels of bilirubin.^16^

## Conclusion

In the present study, we observed that increased levels of bilirubin is associated with reduced survival time and that the level of bilirubin can be used as a costless and easily detectable prognostic marker for overall survival in patients with parotid gland carcinoma. As parotid gland carcinoma is a rare disease, the importance of bilirubin should be supported with further studies.

## Conflicts of interest

The authors declare no conflicts of interest.
